# The Impact of COVID-19-Related Shutdown Measures on the Training Habits and Perceptions of Athletes in the United States: A Brief Research Report

**DOI:** 10.3389/fspor.2020.623068

**Published:** 2020-12-23

**Authors:** Andrew R. Jagim, Joel Luedke, Austin Fitzpatrick, Greg Winkelman, Jacob L. Erickson, Andrew T. Askow, Clayton L. Camic

**Affiliations:** ^1^Sports Medicine, Mayo Clinic Health System, La Crosse, WI, United States; ^2^University of Wisconsin-La Crosse, La Crosse, WI, United States; ^3^Mississippi State University, Starkville, MS, United States; ^4^Department of Kinesiology and Community Health, University of Illinois at Urbana-Champaign, Urbana, IL, United States; ^5^Kinesiology and Physical Education, Northern Illinois University, DeKalb, IL, United States

**Keywords:** coronavirus, strength and conditioning, sports, deconditioning, deconditioned

## Abstract

The purpose of the current study was to examine the impact of COVID-19 government-enforced shutdown measures on the training habits and perceptions of athletes. A web-based electronic survey was developed and distributed online to athletes. The survey contained questions regarding currently available resources, changes in weekly training habits, and perceptions of training such as intensity, motivation, and enjoyment. A total of 105 (males: *n* = 31; females: *n* = 74) athletes completed the survey (mean ± *SD* age = 19.86 ± 2.13 years). Ninety-nine (94.3%) athletes continued to receive guidance from their primary sport coach or strength training staff. There was a significant (*p* < 0.001) decrease (mean ± *SD*) in self-reported participation time for strength training (−1.65 ± 4.32 h. week^−1^), endurance (−1.47 ± 3.93 h. week^−1^), and mobility (−1.09 ± 2.24 h. week^−1^), with the largest reduction coming from participation time in sport-specific activities (−6.44 ± 6.28 h. week^−1^) pre- to post-shutdown. When asked to rate their current state of emotional well-being using a visual analog scale of 0–100, with 100 being exceptional, the mean score was 51.6 ± 19.6 AU. Athletes experienced notable reductions in training frequency and time spent completing various training related activities. In the future, practitioners should have preparations in place in the event of another lockdown period or future pandemic to avoid or minimize significant disruptions in training. Special considerations may be needed when athletes are allowed to return to sport in the event of significant levels of detraining that may have occurred.

## Introduction

In 2020, the sports world was halted by a global pandemic that resulted in a stoppage of most athletic competitions. The Severe Acute Respiratory Syndrome Coronavirus 2 (SARS-CoV-2) responsible for the respiratory condition known more commonly as COVID-19 can result in pneumonia-like symptoms and may elicit an inflammatory cascade impacting the bronchioles, alveoli, cardiovascular system, and myocardial tissue (Buscarini et al., 2020; Cipollaro et al., 2020; Ding et al., 2020; Lovato and De Filippis, 2020). In March of 2020, the World Health Organization (WHO) classified COVID-19 as a global pandemic (World Health Organization, 2020). In an effort to prevent community transmission and to “flatten the curve” (in reference to reducing large spikes in positive cases and potential for high in-patient volumes), national- and state-level officials within the United States (US) implemented several restrictions and safeguards. These precautionary measures included social distancing, restrictions on public gatherings, and stay-at-home orders for several states within the US, which resulted in the closure of most non-essential businesses. Included in this closure were most athletic training facilities and community fitness centers which prevented athletes from accessing strength and conditioning equipment. As a result, athletes had to significantly modify their training habits and environment without adequate time to secure appropriate training equipment.

Until June of 2020, the majority of athletes across all levels of competition within the US were still not able to compete in organized sporting events or in-person training activities due to the continued health concerns of COVID-19 and incumbent precautionary measures. Over the summer of 2020, some professional sports organizations such as the National Basketball Association, Professional Golf Association, and eventually Major League Baseball were able to return to competition albeit using extreme precautionary measures such as complete isolation of athletes and staff members in a “bubble” environment or the sport naturally lent itself to easy isolation of athletes resulting in a seemingly safer sport environment with a higher chance of success. However, this was not a financially viable option for smaller leagues or those competing at the college and high school level. Therefore, smaller leagues continued to prohibit in-person sport-related activities through most of the summer of 2020 and some have even differed competition through the end of the year in some cases.

The COVID-19 pandemic response resulted in an unprecedented situation within the sporting community. Resultantly, athletes were forced to abruptly modify training habits without adequate time for coaches, strength, and conditioning professionals and athletes to develop a structured strategy moving forward. While previous commentaries (Sarto et al., 2020) and consensus statements have been recently published regarding concerns for athletes returning to sport after a disruption in regular training or recommendations for in-home training (Hammami et al., 2020; Jukic et al., 2020), there are currently a lack of data available that has examined the specific changes of athletes' training habits. Therefore, the purpose of this brief research report was to examine the effect that COVID-19 government mandated shutdown measures had on the training habits and perceptions of athletes within the US.

## Methods

### Design

This cross-sectional study design distributed an electronic survey to a mixed-sex cohort of athletes. The survey was designed to examine how the COVID-19 pandemic during the spring of 2020 and the ensuing response from sporting organizations and government-mandated shutdown measures impacted the training habits, perceptions of effort, and motivation of athletes within the United States. The survey was distributed between April 2020 and June 2020.

### Participants

A total of 136 athletes initially responded to the survey with 105 (males: *n* = 31; females: *n* = 74) of these individuals (mean ± *SD* age = 19.9 ± 2.1 years; weight = 76.1 ± 23.7 kg.; height = 170.6 ± 11.6 cm) completing the survey. Of those who completed the survey, 92 (87.6%), 10 (9.5%), and 3 (2.9%) of the athletes competed at the collegiate, high school, or “other” level of sport, respectively. Athletes from the following sports were represented: football (*n* = 21), baseball/softball (*n* = 22), soccer (*n* = 10), track/cross country (*n* = 17), volleyball (*n* = 2), basketball (*n* = 8), lacrosse (*n* = 5), gymnastics (*n* = 16), golf (*n* = 3), and rugby (*n* = 1). Participants were informed of the study purpose, details of their participation, and target outcomes prior to providing electronic consent at the start of the survey before being allowed to continue. This survey was approved by the Institutional Review Board of the University of Wisconsin—La Crosse (IRB# 20-AJ-388 5/22/2020) in compliance with the Human Subjects Guidelines for Research.

### Data Collection Procedure

An electronic survey (Qualtrics, Provo, UT, USA) was distributed via email and social media, targeting practitioners, athletic trainers, strength staff, and coaches to further distribute the survey to their athletes. The survey consisted of 36 questions that were designed to assess basic demographic information (*n* = 10), the current training-related resources and programming available (*n* = 8), changes in training habits (*n* = 12), and how their perceptions of effort, enjoyment from training, motivation to train, and perception of overall mental health changed, as a result of the COVID-19-related shutdown measures (*n* = 6). An anonymous link to the survey was publically posted on social media for sharing and redistribution. Additional participants were sent emails with an accompanying link from their athletics staff or coaches on behalf of select institutions within the United States who were asked to participate in the study through word of mouth.

### Statistical Analysis

The data were analyzed using descriptive statistics to summarize participant demographics and current resource changes in training habits and perceptions. Paired sample t tests were used to assess changes in training habits. All data are presented as means ± standard deviation with 95% confidence intervals. Statistical significance was determined at *p* < 0.05. All data were analyzed using SPSS V.25 (IBM Corporation, Armonk, NY, USA).

## Results

### Athlete Background and Resources

Seventy (66.7%) athletes reported being actively participating in a winter or spring sport that was canceled due to the COVID-19 shutdown measures. Eighty-eight (83.8%) athletes reported that they were completing current training activities alone. Ninety-nine (94.3%) athletes continued to receive guidance from their primary sport coach or strength training staff. A summary of the equipment available to the survey respondents at the time of survey completion is presented in Table 1.

**Table 1:** A summary of equipment available to athletes at home.

**Available equipment**	***n*= 105**	**%**
Resistance bands	65	61.9
Kettlebells	22	21.0
Dumbbells	61	58.1
Barbells and plates	32	30.5
Stationary machines	23	21.9
Other	16	15.2

### Training Habits

A summary of self-reported training goals during COVID-19 related shutdown period are presented in Table 2. Notable reductions in overall training frequency were reported during the COVID-19-related shutdown period. Pre-COVID-19, 83 (79%) athletes reported training 5–6 days per week compared to only 48 (45.7%) during the COVID-19-related shutdown period. A complete summary of changes in training frequency are presented in Table 3. There was a significant (*p* < 0.001) decrease (mean ± *SD*) in self-reported participation time for strength training (−1.65 ± 4.32 h · wk^−1^), endurance (−1.47 ± 3.93 h · wk^−1^), and mobility (−1.09 ± 2.24 h · wk^−1^), with the largest reduction coming from participation time in sport-specific activities (−6.44 ± 6.28 h · wk^−1^) pre- to post-shutdown. Changes in time spent completing specific styles of training are presented in Table 4. Female athletes self-reported a greater decrease in time spent doing sport-specific (females: −8.16 ± 5.12; males: −2.79 ± 6.41 h; *p* < 0.001) and mobility training (females: −1.52 ± 2.21; males: −0.30 ± 1.66 h; *p* < 0.01) compared to males.

**Table 2:** Frequency of self-reported training goal during COVID-19 related shutdown period.

**Self-reported training goal**	***n* = 105**	***%***
Strength	21	20
Muscular endurance	8	7.6
Muscle growth	8	7.6
Increase weight	2	1.9
Lose weight	12	11.4
Maintain weight	4	3.8
Training for competition/sport	38	36.2
General health	5	4.8
Stress management	3	2.9
Enjoyment/leisure activity	4	3.8

**Table 3:** Changes in training frequency.

**Training frequency**	**Pre-COVID-19**	**Currently**
	***n* (%)**	***n* (%)**
1 day per week	0 (0)	11 (10.5%)
2 days per week	2 (1.9%)	12 (11.4%)
3 days per week	4 (3.8%)	13 (12.4%)
4 days per week	10 (9.5%)	17 (16.2%)
5 days per week	46 (43.8%)	25 (23.8%)
6 days per week	37 (35.2%)	23 (21.9%)
7 days per week	6 (5.7%)	4 (3.8%)

**Table 4:** Changes in time spent on specific styles of training.

**Training time per week (hrs.)**	**Pre-COVID-19**	**Currently**	**Delta**	**95% CI**	***p*-value**
Strength training	6.88 ± 4.37	5.22 ± 3.73	1.65 ± 4.32	0.81, 2.49	<0.001
Conditioning activities	6.45 ± 4.16	4.98 ± 3.19	1.47 ± 3.93	0.71, 2.24	<0.001
Mobility / flexibility	3.80 ± 2.53	2.70 ± 2.15	1.09 ± 2.24	0.66, 1.53	<0.001
Sport-specific activities	10.51 ± 5.59	4.07 ± 3.59	6.44 ± 6.28	5.19, 7.69	<0.001

### Perceptions of Training and Mental Well-Being

Seventy-one (67.6%) athletes reported a decrease in the level of motivation to train compared to pre-COVID-19 times, while 18 (17.1%) reported the same level of motivation to train. Sixty-nine (65.7%) athletes reported lower training satisfaction scores while only 14 (13.3%) reported the same levels of training satisfaction, compared to pre-COVID-19 times. When asked to rate their current state of emotional well-being using a visual analog scale of 0–100, the mean score was 51.59 ± 19.59 AU.

## Discussion

In 2020, the world was faced with a global pandemic that resulted in an unprecedented disruption in sport. The resulting impact on the specific training habits and perceptions of athletes has yet to be elucidated. The primary purpose of the current study was to investigate the effects of the US government-mandated COVID-19 shutdown measures on the training habits and perceptions of athletes. The principle findings of the current study were that athletes self-reported significant changes to their training frequency, time spent doing training activities, motivation to train, enjoyment from training, training effort, and dietary habits compared to pre-COVID-19 activities. Specifically, the survey results indicated that those training 5–6 days per week dropped from 79% of respondents prior to COVID-19 to 46% at the time of survey completion. However, none of the athletes reported a complete cessation from training during the pandemic. There were significant decreases in the amount of self-reported weekly training time dedicated to strength, conditioning, and mobility activities (Table 4). The largest such change in training habits occurred in relation to the time spent completing sport-specific activities, in which there was a decrease of ~6.4 weekly training hours from pre- to post-shutdown, which was likely attributed to the cessation of in-person organized team practices as part of the COVID-19 shutdown measures.

The most frequently reported training goal during the COVID-19 shutdown period was “training for competition and sport” at 36.2% and “training for strength” at 20%. Training for weight loss (11.4%), weight gain (1.9%), stress management (2.9%), and general health (4.8%) were less common. Additionally, the majority of the athletes self-reported making a change to their dietary habits as an adjustment to their altered training. The majority of the athletes surveyed (94.3%) reported receiving continued guidance from their respective strength and conditioning professionals or sport coaches, which is noteworthy as 83.8% of athletes reported completing training activities alone. These findings demonstrate the importance of continued communication between athletes and strength and conditioning professionals in the event of in-person activities not being permitted. As a result of the COVID-19 shutdown measures, many of the athletes had to complete training on their own, likely in unique environments with varying resources. Nearly half of the athletes in the current study reported having access to resistance bands and dumbbell weights, whereas very few had access to barbells or kettlebells. Therefore, without any degree of consistency across players, strength staff were likely faced with numerous challenges when trying to implement appropriate training programs with any degree of cohesiveness or structured periodization scheme. This lack of resources and preparation was likely exacerbated when little notice of such a drastic transition, as with the current situation with COVID-19, was provided, thus limiting athletes' ability to make adequate preparations ahead of time and their ability to follow a structured training program to help facilitate the appropriate progression of regular adaptations to training.

Collectively, disruption in normal sport activities and prohibition of in-person activities may influence an athlete's perception of and willingness to train. When athletes in the current survey were asked to rate their training motivation, 68% reported a decrease while only 17.1% reported the same level of motivation to train during the COVID-19 shutdown period compared to pre-COVID-19 times. Athletes also reported decreases in training satisfaction as 65.7% reported lower training satisfaction scores while only 13.3% reported the same levels of training satisfaction. Furthermore, the majority of survey participants who reported training at lower intensities compared to pre-COVID-19 shutdown measures took effect. Albeit subjective, this self-reported decrease in perceived training intensity could diminish an athlete's state of physical readiness when a return to sport is possible. However, the downstream and long-term effects of these disruptions in sport activities and training will not be fully elucidated for several years to come. It is likely safe to presume that a lack of in-person support and instruction from coaches, strength staff, and athletic trainers would have a detrimental impact on the quality and safety of training and sport-specific activities, certainly as it pertains to tactical strategies, team chemistry, and game flow. Further, the lack of interaction with team members is likely to negatively influence the mental health of athletes while perpetuating feelings of isolation, lack of motivation, and desire for in-person socialization. Athletes in the current study self-reported their overall state of mental well-being to be approximately at a score of 50, or “average” on a scale of 0–100.

Another major concern stemming from disruptions in proper physical training for sport is the increased risk of future injuries when athletes return to play. Myer et al. (2011) reported a 4-fold increase in Achilles tendon ruptures during the pre-season period in National Football League players following a player lockout when compared to the average number of ruptures during the preceding five seasons. The authors speculated that a lack of an adequate preparatory strength and conditioning period likely resulted in a detraining effect, thus predisposing athletes to the greater risk of soft-tissue injuries during explosive activities (Myer et al., 2011). Therefore, as athletes return to competition in the 2020–2021 season, there may be an increased incidence of sports-related injuries; however, future observational research is needed to support this hypothesis. While not necessarily a direct risk to athlete health, extended periods of detraining are also likely to result in several physiological changes, including reductions in strength, power, speed, and endurance (Mujika and Padilla, 2000a,b; Ingle et al., 2006; Izquierdo et al., 2007; Ormsbee and Arciero, 2012; Melchiorri et al., 2014), which may have a subsequent impact on sport performance and may also play a role in the aforementioned increased risk of injury. For example, Ormsbee and Arciero (2012) reported significant increases in body fat percentage and body weight with a concomitant decrease in aerobic capacity after only 5 weeks of detraining following a competitive season. Recently, Muriel et al. (2020) were the first to quantify objective measures of training load during the COVID-19 lockdown period in elite cyclists. The authors noted 34% reductions in total training volume and reductions in weekly volumes across intensity zones ranging from 25 to 52% (effect size: 0.83–1.57) during the lockdown period, which contributed to reductions in maximal effort cycling performance (Muriel et al., 2020). Similarly, Zinner et al. (2020) observed reductions in overall weekly training time (−27.6%) and the average length of training sessions (−15.4%) during a COVID-19 lockdown period in highly trained German kayakers and canoeists. The authors also observed an increase in the number of strength-focused training sessions with no change in sport-specific activities, which is contradictory to the findings from the current study (Zinner et al., 2020). It is possible that athletes at the elite level may have had better access to strength equipment, thereby allowing for increased opportunities for strength workouts. While a brief period of rest or detraining at certain time points throughout an annual training cycle is common practice, the unprecedented cessation of certain sport activities and robust disruption of training experienced by most athletes in 2020 will likely lead to notable decrements in training adaptations similar to those noted by Muriel et al. (2020). Further, the specific decreases in performance-related measures may be more pronounced in athletes lacking adequate home-based training equipment or those unable to complete sport-specific activities. There have been several sport-specific return-to-play guidelines (Elliott et al., 2020; Jukic et al., 2020; Mohr et al., 2020; Sarto et al., 2020) recently published that outline progressive and sport-specific methods to slowly re-condition athletes and prepare them for future competitions, which will be essential for coaching staff to be cognizant of. Additionally, there are also commentaries available regarding special considerations for athletes returning to sport post COVID-19 infection (Dores and Cardim, 2020; Elliott et al., 2020), which is another major concern of the current pandemic. Because of the novelty of this virus, there is a paucity of data available regarding the long-term physiological effects of infection, which has resulted in a high level of concern from sports medicine professionals regarding the decision to allow sporting activities to resume and when or how to clear athletes to a return-to-play post-infection.

## Limitations

We note several limitations of this study. First, the survey period was cut short because of the loosening of government-mandated shutdown measures, which allowed some athletes to return to in-person training activities. Thus, a limited sample size was included in the final analysis. The majority of responses received for our survey came from NCAA Division III collegiate athletes during the onset of the COVID-19 pandemic, which increases the risk of selection bias and subsequent relevance and generalizability to athletes in different settings (i.e., NCAA Division I collegiate, and professional athletes). As such, the athletes in the current study likely have differing levels of access to training modalities and resources, including certified strength and conditioning professionals that may be more readily available at higher levels of competition or those with more financial resources. Another limitation is the use of self-reported measures of training time to quantify changes in training activities rather than objective measures of training load or volume. More controlled methods of training load monitoring could have helped discern specific modifications that were made to the training programs of each cohort of athletes. Further, pre-COVID-19 measures of performance would have allowed for more precise insight regarding how the current state of detraining would have impacted measures of strength, power, endurance, and sport-specific tasks. Lastly, the lack of open-ended survey questions reduce the ability for athletes to elaborate on how COVID-19 impacted their perceptions of training or what contextual factors may have also contributed to the observed changes in training.

## Conclusions

Although likely rare, the field can learn from this unprecedented stoppage in sport and seek strategies to better prepare in the event of future pandemics of this nature. Results of the current study indicate that athletes did not have a diverse array of resistance training equipment available to them at the time of the shutdown measures. Additionally, athletes reported notable reductions in training frequency and time spent on sport-specific training activities. An important caveat to the current situation is that athletes and coaches did not have adequate time to appropriately prepare training programs designed for in-home training, especially for an extended period of time, or to secure appropriate equipment for home-based training. As the current pandemic situation continues to evolve, or in the event of another global pandemic, athletes and strength staff can learn from this situation to have contingency plans in place if home-based training is needed again in the future. Moving forward, strength and conditioning professionals should prepare in-home training program options and look to integrate easy-to-implement training modalities (i.e., body weight, bands, kettlebells, plyometrics, etc.), to elicit the desired training stress required to optimize or at least maintain training adaptations in the event of another shutdown period. Additionally, athletic departments may consider investing in mobile training equipment kits that could be checked out to athletes in the event of extended periods of lockdown. The ability to maintain training adaptations and conditioning levels during a pandemic or lockdown would likely contribute to increased injury resilience and a safe return to sport following an extended period of decreased physical activity.

## Data Availability Statement

The raw data supporting the conclusions of this article will be made available by the authors, without undue reservation.

## Ethics Statement

The studies involving human participants were reviewed and approved by Institutional Review Board for the Protection of Human Subjects at the University of Wisconsin-La Crosse. The study was conducted according to the Declaration of Helsinki guidelines. The patients/participants provided their written informed consent to participate in this study.

## Author Contributions

AJ, JL, CC, and AA: conceptualization. AJ, JL, JE, and AA: methodology. AJ, AA, and CC: formal analysis. AJ, JL, AF, GW, and AA: writing—original draft preparation. AJ, JL, AF, GW, JE, AA, and CC: writing—review and editing. All authors have read and agreed to the published version of the manuscript. All authors contributed to the article and approved the submitted version.

## Conflict of Interest

The authors declare that the research was conducted in the absence of any commercial or financial relationships that could be construed as a potential conflict of interest.
